# Supplementary material for: The Construction and Exploration of a Comprehensive MicroRNA Centered Regulatory Network in Foxtail Millet (Setaria italica L.)
Source: Front Plant Sci. 2022 May 6;13:848474. doi: 10.3389/fpls.2022.848474 (PMC9121102; doi:10.3389/fpls.2022.848474)
Supplement: Supplementary file 3 [file Data_Sheet_3.zip › Sit-miR171g_Seita.5G018900.1_165_TPlot.pdf]

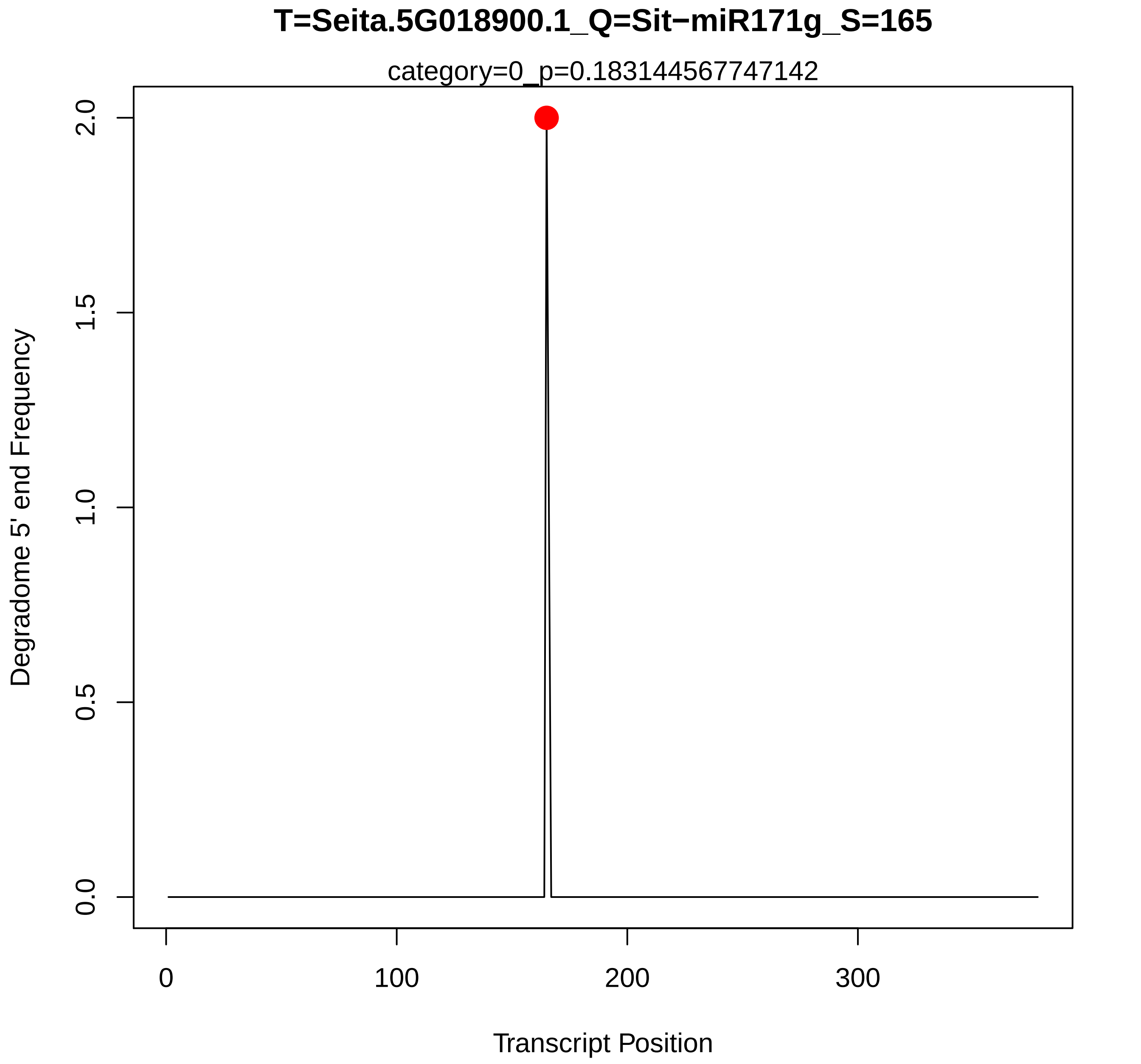

T=Seita.5G018900.1_Q=Sit−miR171g_S=165
category=0_p=0.183144567747142
Degradome 5' end Frequency
2.0
1.5
1.0
0.5
0.0
0
100
200
300
Transcript Position
